# Job-Related Stress in Forensic Interviewers of Children with Use of Therapy Dogs Compared with Facility Dogs or No Dogs

**DOI:** 10.3389/fvets.2018.00046

**Published:** 2018-03-12

**Authors:** Diane Walsh, Mariko Yamamoto, Neil H. Willits, Lynette A. Hart

**Affiliations:** ^1^School of Veterinary Medicine, University of California, Davis, Davis, CA, United States; ^2^Department of Animal Sciences, Teikyo University of Science, Uenohara, Japan; ^3^Statistical Laboratory, University of California, Davis, Davis, CA, United States

**Keywords:** avoidance, courthouse dogs, facility dogs, forensic interviews, secondary traumatic stress, therapy dogs, secondary traumatic stress scale

## Abstract

Sexually abused children providing essential testimony regarding crimes in forensic interviews now sometimes are provided facility dogs or therapy dogs for comfort. Facility dogs are extensively trained to work with forensic interviewers; when using therapy dogs in interviews, volunteers are the dog handlers. Interviews can impact child welfare workers’ mental health causing secondary traumatic stress (STS). To investigate this stress, first data were gathered on stress retrospectively for when interviewers initially started the job prior to working with a dog, and then currently, from forensic interviewers using a facility dog, a therapy or pet dog, or no dog. These retrospective and secondary traumatic stress scale (STSS) data compared job stress among interviewers of children using: a certified, workplace facility dog (*n* = 16), a volunteer’s trained therapy dog or the interviewer’s pet dog (*n* = 13/3), or no dog (*n* = 198). Retrospective scores of therapy dog and no dog interviewers’ stress were highest for the first interviewing year 1 and then declined. Extremely or very stressful retrospective scores differed among the three groups in year 1 (*p* < 0.038), and were significantly elevated for the therapy dog group as compared with the facility dog group (*p* < 0.035). All interviewing groups had elevated STSS scores; when compared with other healthcare groups that have been studied, sub-scores were especially high for Avoidance: a psychological coping mechanism to avoid dealing with a stressor. STSS scores differed among groups (*p* < 0.016), primarily due to Avoidance sub-scores (*p* < 0.009), reflecting higher Avoidance scores for therapy dog users than no dog users (*p* < 0.009). Facility dog users more consistently used dogs during interviews and conducted more interviews than therapy/pet dog users; both groups favored using dogs. Interviewers currently working with therapy dogs accompanied by their volunteers reported they had experienced heightened stress when they began their jobs; their high stress levels still persisted, indicating lower inherent coping skills and perhaps greater empathy among interviewers who later self-selected to work with therapy dogs. Results reveal extreme avoidant stress for interviewers witnessing children who are suffering and their differing coping approaches.

## Introduction

At least four million children are abused physically and sexually each year in the United States (1). Prior to 1985, children entering the criminal justice system often encountered great emotional distress during their interviews by detectives, attorneys, or forensic interviewers in a courthouse or police station, with little support from social workers and other child protective services. Child Advocacy Center (CAC) models were developed in the 1980s, bringing a team approach to helping children, combining social workers, forensic interviewers, medical, and mental health workers in a comfortable child-friendly setting. Today, over 800 CACs operate in the United States, serving over 300,000 children per year (2). Children’s statements are often the only evidence available in abuse situations, since the nature of the crime precludes an eyewitness. The forensic interview can be considered the single most important tool in protecting the child and removing the abusers from society. A forensic interview is a structured conversation using established protocols with a child who has allegedly been abused, to draw out detailed information for a criminal investigation about possible traumatic events that the child either experienced or witnessed (3). The emotional demands of this type of work, repeatedly hearing traumatic accounts, can cause stress for the forensic interviewer.

Secondary traumatic stress (STS), also termed vicarious trauma, burnout, or compassion fatigue (4), may occur when professionals, such as child protection workers, child advocates, or forensic interviewers experience stress when working with abused children and their families. Such stress occurs broadly among healthcare providers of traumatized patients and sometimes is described as a cost of caring (5). Among various sources of stress for these workers, the obvious source of stress would be the cumulative effects of hearing accounts of horrific abuse from children daily. But, additional combined stresses of legal liability, long hours, and high workloads (6) further contribute to work-related stress in child protection fields. In a study done on veteran child protection service workers, 62% of the 151 participants scored in the high range on the emotional exhaustion scale, despite the use of a number of coping strategies and tools that were available (6). A meta-analysis of STSS among professionals doing therapeutic work with trauma victims found some statistical significance for trauma caseload volume and frequency and negative effects for work support and social support (4). Another study found that working in child welfare is more likely to predict compassion fatigue and burnout compared to all other types of behavioral healthcare professionals (7). Unlike several other studies, this one reported higher levels of burnout among men, however, men were overrepresented in the sample among the child welfare workers. Vicarious stress arises in the job that is described as high demand/low reward; research indicates that job stress in these types of situations can seriously affect the mental health of the workers (8).

Extreme job stress and burnout has been studied among child welfare workers and oncology nurses. Although burnout is considered to emerge with prolonged job stress from emotional exhaustion and depersonalization, whereas STS can come on suddenly (9), data on well-studied burnout may somewhat overlap with the stresses of forensic interviewing. The U.S. annual turnover rate in child welfare work, another stressful and similar type of work with children, is 30–40%, with a mean stay of 2–4 years (10); this accounts for focused research on child welfare workers. A study of 209 New England child welfare workers explored the roles of job stress, burnout, and emotional exhaustion as they contributed to an intention to leave the job; protective factors for planning to stay in the workplace included age, having children, and having a management position (10). Emotional exhaustion, disengagement from work, and depersonalization (distancing from clients) often are the focus of studies regarding burnout. A recent burnout instrument, the Oldenburg Burnout Inventory, included measures of the positive opposites of exhaustion and disengagement, termed vigor and dedication (11). They found that healthcare professionals are at greater risk for burnout than white-collar professionals.

In practice, a child forensic interviewer uses a number of investigative processes when interviewing a child who has allegedly been abused to determine whether abuse has actually occurred and, if so, obtain information that can be used in court proceedings. The interviewer must remain a neutral party while collecting evidence in an alleged crime.

Videotaped forensic interviews sometimes are allowed to be introduced into court as hearsay testimonial evidence, however, since the 2006 U.S. Supreme Court ruling of Crawford v. Washington it has generally been required that the defendant be able to confront the accuser in court; yet, extensive legal argument is also made that the perpetrator has relinquished the right to confront the young vulnerable victim (12, 13). The forensic interviewer is not allowed to do anything that would lead a child to a certain conclusion, express sympathy, or provide comfort through a touch or voice (14). The demands of remaining a neutral party while intimately engaged in the lives of children who sometimes relay appalling details of abuse can be a significant source of stress. Over time this stress can increase exhaustion, cynicism, and detachment from the job of forensic interviewing (15).

Forensic interviewers employ various techniques to help in relieve stress of the children they are interviewing, as described in the industry standard, the National Institute of Child Health and Human Development Investigative Interview Protocol (16). Additional techniques are used, such as anatomical dolls, questions with directives using free recall (17), and having a child use drawings to tell the story of what happened to them (16).

An expanding innovation in the forensic interview is incorporating a certified facility dog that is given an extensive 2-year training program specific to the forensic interviewing process (18). Canine Companions for Independence [CCI (19)] has trained most of these dogs, using similar methods as when training service dogs. The number of these facility dogs that had been placed for forensic interviewing by September 2014 when this study was done was 70, and by 2016, 127 had been placed; not all of these dogs were yet actively involved in forensic interviews (Celeste Walsen, private communication). The facility dogs work with employed child advocates or court personnel to provide emotional support to the child that the forensic interviewer is unable to provide, since legal neutrality is the crucial component of the forensic interview. Because of their training and behavior, a certified facility dog incorporated into the forensic interview process may be an extremely valuable tool for the investigation of crimes and result in a higher quality investigation. As defined by Pet Partners (20), facility dogs are specially trained to be regularly present in a clinical setting, living with an employee of the facility or at the facility with care by a staff person.

Some CACs use therapy dogs, or even pet dogs (usually belonging to someone at the center), for interviews rather than trained facility dogs. As defined by Pet Partners (20), therapy dogs provide affection and comfort to members of the public and their owners volunteer their time to visit with their animals. For forensic interviews, the volunteer brings the therapy dog into the office for the interview upon request, whereas a facility dog is a member of the office staff that works daily in the center where it is available. Centers may require that therapy dogs be tested for temperament and obedience by a National Organizations, such as Pet Partners (20), or further trained for use in therapy with adult and child patients. Unlike facility dogs that assist a professional employee, therapy dogs are not trained to perform specific tasks. The CAC interviewer arranges scheduling and then deals with the volunteer who brings in the therapy dog. A small number of dogs performing as therapy dogs are pet dogs that have not been through behavior testing, but are well trained with mild temperaments. For confidentiality reasons, the handler is not allowed to hear evidence in a criminal case; the handler either hands the dog over to the interviewer or keeps a long lead on the dog and stands outside of the interview room holding the lead, or may wear earphones to prevent hearing the interview.

Differences between facility and therapy dogs may not be evident to children, whose experiences may be similar in both cases, having a dog to hold and pet while testifying. From the perspective of the International Association of Human-Animal Interaction Associations, both involve a team that is led by a professional and are considered to be animal-assisted interventions (21). The forensic interviewers’ experience differences with the two types of dogs; with a facility dog, the interviewer is usually an employee of the legal system who is regularly with the dog in the workplace and may also take the dog home as its caregiver. With a therapy dog, the primary handler of the dog is a volunteer who is contacted to bring and manage the dog at the courthouse or CAC and is also likely to be present during the interview or testimony. A few interviewers may bring in their pet dogs to assist in interviews.

This study gathered self-reported data from forensic interviewers, using an anonymous web survey comparing the retrospective and current job-related stress of interviewers who currently used specifically trained facility dogs, therapy or pet dogs, or no dogs. A central question was: does the presence of a facility dog or therapy dog affect the stress of forensic interviewers? Specific research goals included: (1) assess stress levels of all interviewers during their initial years of interviewing (prior to acquiring a dog), using retrospective questions; and (2) assess the current level of stress during the previous week using the Secondary Traumatic Stress Scale (STSS). When dogs were used, the survey also collected self-reported data on the interviewers’ perceptions about how the dogs fit into their practice.

## Materials and Methods

### Sampling and Data Collection

Forensic interviewers working in CACs were recruited as subjects for this study. A regional director in the national CAC organization assisted by posting a message on a listserv for forensic interviewers in the U.S., providing a link to the survey. While approximately 700 people subscribed to the National Children’s Alliance for the CACs listserv at that time, we cannot judge how many persons actually read the message. This listserv email included a brief introduction to the study. The participants responded anonymously and were blind to the research questions regarding secondary stress in interviews and the role that dogs may play in the level of stress. The message was posted twice to the listserv within a 4-week period in September 2014 to give forensic interviewers a reminder.

Using the online web SurveyMonkey survey, data were gathered concerning interviewers’ initial experiences as they began their work as interviewers, including only data prior to the interviewer using a dog. Current stress was assessed using the STSS (22). The web survey data compared job stress among forensic interviewers who used: a certified facility dog working with an employee (*n* = 16); a trained therapy dog brought by a volunteer (*n* = 13); a pet dog associated with the interviewer or colleague at work brought in for the animal-assisted activity (*n* = 3); or no dog (*n* = 198).

### Questionnaire

#### Demographic Information

Questions on simple demographics pertained to marital status, age range, gender, and number of years interviewing.

#### Retrospective Self-Reporting of Work-Related Stress

All interviewers were asked to rate their stress levels on a 5-point Likert scale for each year of their careers from year 1 to year 5; these data were only included for years prior to any use of a courthouse dog.

#### Secondary Traumatic Stress Scale

The STSS is an instrument for assessing STS, a syndrome almost entirely consistent with Post Traumatic Stress Disorder (PTSD), as described in the *Diagnostic and Statistical Manual of Mental Disorders* (23). The fifth edition of this manual (24) recently has further clarified that secondary exposure to PTSD can lead to the development of impairing symptoms requiring treatment (4). The STSS instrument measures work- related STS in human service professionals who have indirect exposure to traumatic events while working with abused and traumatized individuals (22). The STSS measures three subscales that measure methods of coping with responses to painful past experiences following traumatic stress: intrusion, when certain past memories persist in returning; avoidance, attempting to avoid stimuli that bring back memories of something painful; and arousal, tending to be easily startled or extremely irritable. It consists of 17 questions with multiple-choice answers on a 5-point Likert scale. Each question describes a symptom, designed to tap into the criteria for PTSD, such as “I had trouble sleeping.” Participants rate their symptoms from “never” to “very often.” Using the STSS in our online survey, the subjects indicated how frequently they had experienced each of the 17 symptoms during the previous week. Available procedures for scoring the results of the stress survey include an algorithm method, a percentile method and a direct cut-off method (25). In the direct cut-off method we used, individuals with a score of 38 or higher on the total score of the STSS are considered to have PTSD due to STS that should be addressed (25). The STSS is widely used and has been thoroughly tested through “convergent, discriminant and factorial analyses” and has demonstrated concept validity (22).

### Interviewers with No Dogs

All interviewers were asked what types of tools, such as dolls or anatomical drawings that they used in their profession to help a child describe the abuse they suffered. The last question asked in the “Tools” section was “Do you use a dog in your practice?” Subjects who did not use dogs in their practice were forwarded to the end of the survey where a general question asked what they do in their daily lives to relieve job stress. There was no opportunity to return to the survey after choosing the “no dog” option.

### Interviewers Using Facility Dogs or Therapy/Pet Dogs

Interviewers who used dogs, whether facility, therapy, or pet dogs, were then asked specifically how long they had used dogs in their practice, how often they used the dogs each week, how many interviews they performed each week, if the dog lived with them and, if not, how often they handled the dog. Next, they were asked to rate their own stress levels prior to having a dog and after using a dog in their profession. Interviewers were also given the opportunity in the form of short essays to describe the sources of their stress, how a dog helps or hinders their work, and their perceptions on how the use of a dog helps a child testify compared to other tools they use in their profession.

### Institutional Review Board Approval

The survey was conducted with anonymous and voluntary participation, and the study was approved by the University of California, Davis, Institutional Review Board Protocol #601883-1.

### Statistical Analyses

Statistical analyses included basic descriptive statistics, as well as linear models with Tukey multiple comparisons and mixed logistic models. Responses were transformed as needed to bring the error distribution in line with the assumption of normality (Wilk–Shapiro test, *W* > 0.95). When no transformation was found that normalized the residual errors, Kruskal–Wallis tests were used to compare groups. Fisher exact tests were used to compare retrospective stress levels among groups. A classification tree is presented to clarify the avoidant stress sub-scores. Tree nodes were tested using a series of *t*-tests. To assess relationships among the interviewers’ initial levels of stress when beginning interviews, their current sub-scores on avoidance on the STSS, and working with one of the dog types, mixed model analyses were used, with SAS version 9.4 and R. The extent to which interviewers used dogs and their frequency of interviews were assessed using descriptive statistics.

## Results

### Sample Characteristics

The total sample size was 230 forensic interviewers. Survey respondents were primarily female (85%), married or in a domestic partnership (70%), and had a median age range of 40–49 years.

The majority of respondents (*n* = 198) did not use a dog in their practice. Of the participants using dogs in their interviews (*n* = 32), all had begun their interviewing without dogs and some years later decided to try working with a dog. The two types of dogs had similar numbers: facility dog handlers (*n* = 16) and interviewers using therapy dogs brought in by a volunteer when needed (*n* = 13), as well as three pet dogs with no special preparation, and brought in sporadically as animal-assisted activity dogs (*n* = 3). For these three dogs, one interviewer was the handler, one handled the dog part-time, and one was not the handler; these three interviewers all had a total of 12+ years of experience with interviewing, including their years working without a dog and their later years with a dog.

Interviewers not using dogs had been practicing for a mean of 8 years. All interviewers who later acquired dogs began their professional years interviewing without dogs. Interviewers currently with facility dogs had been practicing for an average of 9 years and had used a facility dog for an average of 1.7 years. Interviewers working with therapy dogs and their handlers had been practicing for an average of 10 years and using dogs in their practice for an average of 3 years.

### Retrospective Self-Reporting—Stress Levels during First 5 Years of Forensic Interviewing

The web survey regarding both retrospective experience and current experience was completed on one occasion. Subjects were asked to recall their initial years of interviewing (only including data prior to interviewers obtaining a dog) and rate their stress levels retrospectively each year for their first 5 years on the job on a Likert scale of 1–5, ranging from “not stressful” to “extremely stressful”.

**Figure 1:**
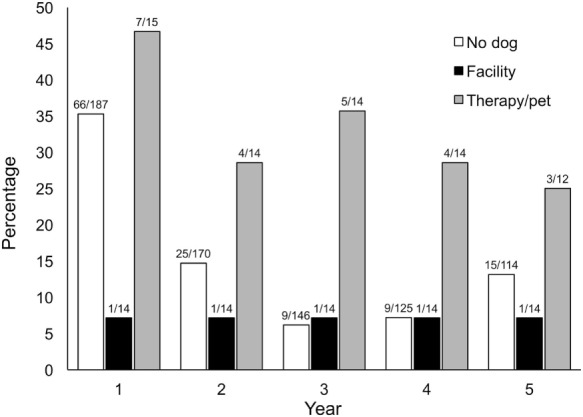
Percentages of no dog, facility dog, and therapy dog groups describing their job stress as “Extremely Stressful” or “Very Stressful” during initial years of interviewing, prior to having a dog. Year 1: three groups—No dog, facility, therapy, *p* = 0.037; two groups—facility vs. therapy, *p* = 0.035.

When considered overall for the 5 years, the three groups did not differ significantly. We then examined data restricted to subjects who had worked more than 5 years and assessed the number of interviewers giving some self-ratings of 4–5 (very stressed to extremely stressed); these results are shown in Figure 1. These stress ratings of 4 or 5 were highest in year 1 for non-dog and therapy dog interviewers and then declined. Facility dog users reported little experience of initial interviewing being very stressful or extremely stressful. The three groups differed in year 1 (*p* = 0.037), with significant differences between the facility and therapy dog groups (*p* = 0.035).

When using logistic models for stress by group to conduct multiple comparisons, based on yes/no responses for high stress, the overall group effect over the 5 years again was not significant (Wald Chi-Square = 4.4; *p* = 0.111). However, in year 1, facility and therapy dog groups again differed: (*z* = −2.1, *p* = 0.036). In year 3 and year 4, no dog and therapy dog groups differed: (*z* = −3.26, *p* = 0.011; *z* = −2.39, *p* = 0.016).

### STSS Results

The STSS scores of all three groups shown in Table 1 indicate STS; some other published comparison figures shown are much lower, from a sample of social workers commonly dealing with patients having secondary stress (25). The total mean score of our respondents who did not use dogs in their practice, 36.9, suggests mild secondary stress, while the mean score for facility dog handlers, 40.3, indicates secondary stress is present, as does the 45.3 score for handlers of therapy dogs. These mean total scores indicate that forensic interviewers in all three groups are subject to STS, ranging from mild to severe.

**Table 1:** Secondary traumatic stress scale mean subscale results.

	Intrusion	Avoidance^c^	Arousal	Total
No dog (*n* = 198)	10.3^a^	15.2^a^	11.4^a^	36.9^a^
Facility dog (*n* = 16)	11.1^a^	16.8^a,b^	12.4^a^	40.3^a,b^
Therapy/pet dog (*n* = 16)	12.3^a^	19.3^b^	13.7^a^	45.3^b^
*p* (group; logs)	0.075	0.009	0.050	0.016
Comparative data from sample of social workers [Bride (25)]	8.18	12.58	8.93	29.69

*Within each column, groups with contrasting letters (^a^ and ^b^) differ significantly*.

*^c^Mann–Whitney U test: p = 0.009, r = 0.18*.

*No dog and therapy dog groups differ after adjusting for first or second year stress, Tukey-Kramer adjustment: p = 0.0001*.

Results on the three subscales for intrusion, avoidance and arousal show no significant differences for the groups of forensic interviewers in the intrusion and arousal sub-scales. However, for the avoidance sub-scale there was a highly significant difference score for persons with therapy dogs as compared with the no dog group and an indication of differences from the facility dog group: parametric analysis of variance on log-transformed data, the dog type (no dog, therapy/pet dog, facility dog) significantly affected the total STSS, *p* = 0.016. A highly significant difference appeared in scores on the avoidance sub-scale, *p* = 0.009, reflecting the elevated stress scores of persons with therapy dogs as compared with no dog interviewers (*p* = 0.009).

To explore the high avoidance scores using GLM analyses in multiple comparisons, we found that even after adjusting for self-reported initial first or second year stress, interviewers with therapy dogs still significantly differed from those with no dogs in currently scoring higher on avoidance (*p* = 0.0001).

**Figure 2:**
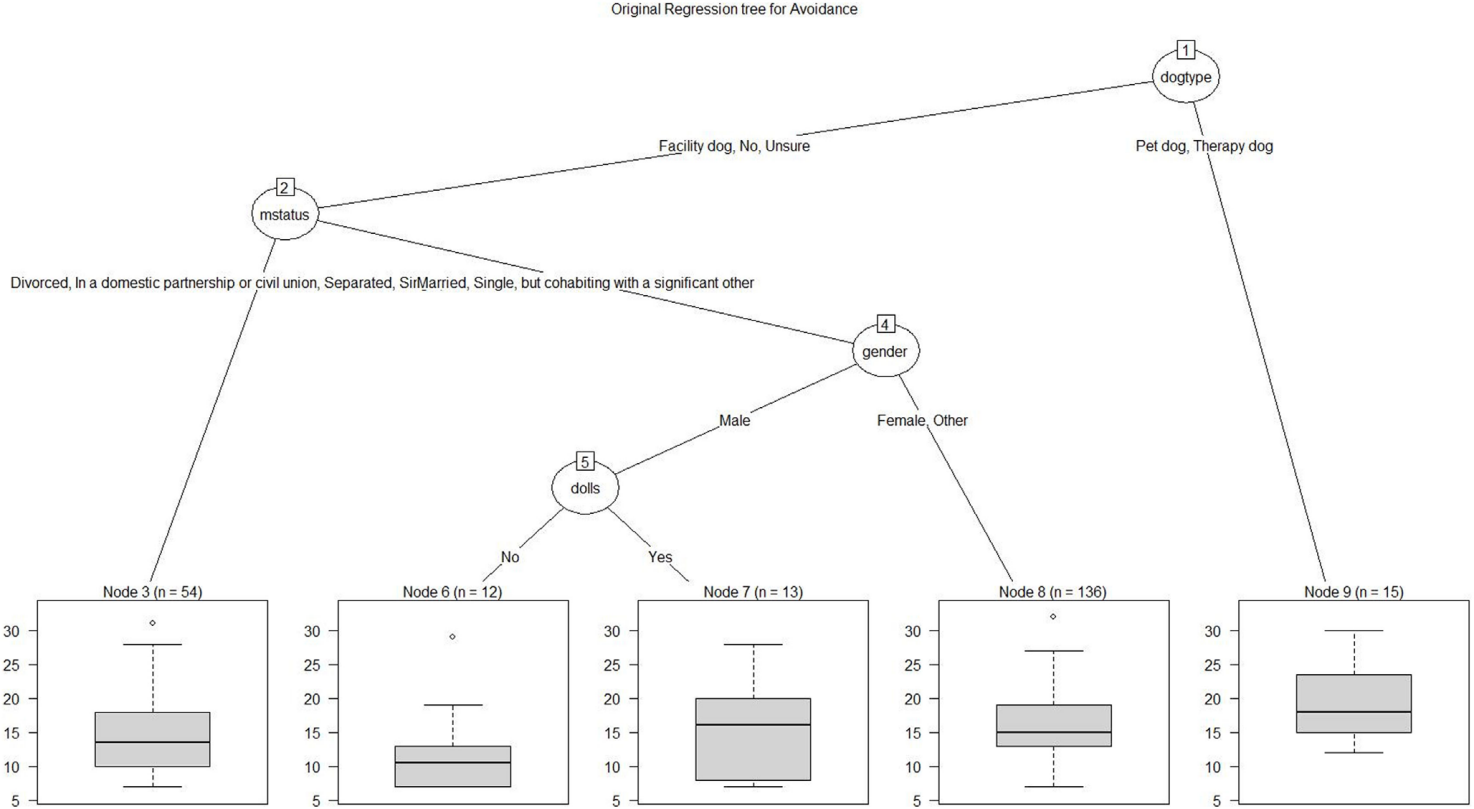
Regression tree plot for avoidance sub-scale scores. Node 1, therapy or pet dog vs. facility or no dog, *p* = 0.004. Node 4, partnered women vs. partnered men, *p* = 0.028.

Further clarifying the extremely high scores on avoidance, a regression tree of the avoidance sub-scale shown in Figure 2 revealed that the dog type was the primary variable, at node 1, with interviewers who used either therapy or pet dogs having elevated avoidance scores, in contrast with those using facility or no dogs. The regression tree indicates that the most elevated avoidance scores were for the 15 participants using therapy or pet dogs in interviews (Mann–Whitney test: *p* = 0.004). The tree split off at node 2 interviewers by marital status, with divorced, single, civil union or single in one group, and married or cohabiting with a significant other in the second group (Mann–Whitney test: *p* = 0.062). The split at node 4 reflected significantly greater avoidance scores for women than men among partnered interviewers (Mann–Whitney test: *p* = 0.028).

### Self-Reporting by Subjects with Facility Dogs or Therapy/Pet Dogs

Facility and therapy groups had differing situations for care of the dog. Six facility dogs had full-time handlers at work that also lived with the dogs, eight had full-time handlers at work that were not living with the dogs, and two had part-time handlers at work that did not live with the dog. The 14 therapy dogs lived with the volunteers who brought them in for interviews and the 2 pet dogs were handled by and lived with interviewers. Facility and therapy/pet dog interviewers significantly differed with regards to spending their time at work with the dogs (*p* < 0.0001). Facility dog and therapy/pet handlers did not differ with regards to living with and handling the dog.

#### Dogs Used in Interviewing Children

All 32 respondents using dogs in their practice were asked to report what they felt, on a Likert scale, about the use of a dog in their practice as compared to the tools they used in their career prior to using a dog, such as anatomical dolls or drawings. The scale ranged from “less helpful,” “just as helpful,” “somewhat more helpful,” “very helpful,” and “extremely more helpful.” None of the respondents felt that a dog was less helpful than other tools in their practice. Seventy-two percent of respondents felt the dog was more helpful, ranging up to extremely more helpful. Twenty-eight percent of respondents thought the use of a dog was just as helpful as other tools, but not more so.

#### Dogs’ Roles in Relieving Work Stress

All participants were asked to rate on a scale of 1–4, the extent to which they felt the use of dogs helped to relieve their own work stress. The scale ranged from “none,” “somewhat,” “very much,” to “immensely.” The results, while statistically non-significant for the two groups, are shown in Table 2. Among all respondents who used either facility or therapy dogs, 75% felt the use of a dog had “somewhat improved” to had an immensely positive effect on their stress. Among interviewers using facility dogs, 94% reported “somewhat” to “immense” relief of stress due to their dog, whereas among interviewers using therapy dogs, 56% reported “somewhat” to “immense” relief of stress due to their dog. A majority of both facility dog (75%) and therapy/pet dog interviewers (66.7%) favored use of dogs.

**Table 2:** Percentage of interviewers reporting dogs changed their work stress.

	No difference (%)	Somewhat improved (%)	Very much to immensely positive (%)
All (*n* = 32)	25	28	47
Facility dog (*n* = 16)	6	31	63
Therapy/pet dog (*n* = 16)	44	25	31

*p > 0.05*.

The number of interviews and the use of dogs in interviews differed between those who used therapy/pet dogs and those who used trained facility dogs. Facility dog handlers performed 6.3 interviews per week, conducting an average of 3.6 interviews per week with the dog. Interviewers who used therapy/pet dogs in their practice averaged 3.3 interviews per week, using the dog in 1.0 interview (number of interviews/week: Kruskal–Wallis test: *p* = 0.011. Our survey did not include a question for the number of interviews performed per week of the participants not using dogs.) Although just a trend, in Table 3, all interviewers using therapy/pet dogs had dogs in their interviews, 50% or less of the time, whereas a majority of the facility dog group used dogs most of the time.

**Table 3:** Percentage of interviews using facility or therapy/pet dogs.

	Percentage				
	0	25	50	75	100
Facility dog (*n* = 16)	12.5	18.8	31.3	25.0	12.5
Therapy/pet dog (*n* = 16)	13.3	53.3	33.3	0.0	0.0

*Mantel–Haenszel Chi-square test, p = 0.08*.

## Discussion

The purpose of this study was to explore the job stress reported by forensic interviewers who use no dogs, facility dogs, or therapy/pet dogs when conducting legal forensic interviews of children. Benefits to the children of using dogs during group therapy were reported in a study of 156 children at a CAC: when dogs were incorporated into the therapy, the children displayed substantial decreases in trauma symptoms (26). More recently, 42 children undergoing forensic interviews involving alleged sexual abuse showed positive effects in salivary immunoglobulin A and heart rates when a therapy dog was used during the interview (27). A further study found elevated heart rates for children not provided a therapy dog during the forensic interview, whereas children with a therapy dog lacked the increase in heart rate (28). In an artificially imposed trauma exposing women to a film, providing a dog was effective in relieving their stress anxiety and anxiety symptoms, but not their physiological stress (29).

Although the effects of dogs on symptoms of secondary stress for interviewers are less studied than direct PTSD, awareness has grown of the secondary stress that human service professionals may suffer due to the sensitive demands of their work. For instance, in social work with children, the incidence of secondary stress is thought to be twice that of the general public (25). Using the STSS to describe their stress during the previous week, a national sample of general social workers reported an average STSS score of 32.07 [*n* = 154 (30)], and a study of emergency nurses in Ireland had an extremely high average score of 45.9 [*n* = 105 (31)]. However, specific to forensic interviewers, a 2012 study of 257 interviewers from across all 50 states found a mean STSS score of 36.7, indicating a mild form of STS in the group studied, that was ameliorated by workplace support [*n* = 256 (32)]. Our no dog group of forensic interviewers scored at a similar level to the mildly stressed forensic interviewers just mentioned, 36.9, but the facility dog and especially therapy dog groups scored at higher stress levels, 40.3 and 45.3, respectively, well above the cut-off score of 38, and reflecting STS that should be addressed.

### Retrospective Self-Reporting of Work-Related Stress

Considering the first five practicing years of the three groups of interviewers, a large portion of the small sample of therapy/pet dog handlers retrospectively reported high stress in their initial years of practice. Only 19% of the therapy dog group reported stress outside of their job (divorce, death, illness) as contributing to their stress on the job. Their heightened stress levels decreased across the initial years, but remained somewhat elevated; this could reflect less effective coping and/or greater empathy. The group with no dogs started interviewing with somewhat elevated stress, but by year 3 their reported stress had lowered.

Also a small sample, facility dog users retrospectively reported little elevated stress across their initial years of interviewing, even though 38% of these respondents reported additional outside stressors that could have contributed to their job stress, such as divorce, death, or illness.

We found heightened levels of self-perceived stress in the initial years for those who later chose to use therapy dogs. Those interviewers later becoming facility dog users, lacking the elevated stress of therapy dog users, perhaps had felt freer to look for the best tool to help their clients, and were more willing to assume the responsibility to be a handler at work with a dog that has been specifically trained for 2 years to work in a legal setting with children. Some facility dog handlers make a commitment to bring the dog into their home and be the primary care provider.

### Secondary Traumatic Stress Scale

Our hypothesis of dogs ameliorating secondary stress for forensic interviewers was not supported by the data collected in the STSS results. Scores for interviewers using therapy/pet dogs indicated significantly even higher, rather than lower, stress than the interviewers with no dogs, largely due to unusually elevated scores on the STSS avoidance subscale. The interviewing inevitably involves repeated experiences with suffering children, perhaps explaining the elevated avoidance scores, as opposed to unwanted recurring memories (intrusion subscale), or feeling extremely aroused or irritable (arousal subscale). Nonetheless, our overall general score results showing elevated STS among forensic interviewers in general were consistent with results from others among social workers dealing with family or sexual violence (30) and among forensic interviewers of children (32). These two earlier studies did not report an effect of gender or the marital status of participants, whereas a tree analysis showed that in this study, no dog, and facility dog interviewers who were married or cohabiting with a significant other had less stress if they were men rather than women.

Witnessing suffering of children is profoundly disturbing to people, as was evident in interviewers’ STSS scores. The extremely high avoidance scores in this study differ from the response profiles of participants in other published studies, and show that these interviewers overall used avoidance as a coping method. For example, they may have responded positively to this STSS item: “I avoided people, places, or things that reminded me of my work with clients.” These participants seem to have become emotionally overloaded, spread thin, and exhausted with their toxic secondarily stressful experiences. A published social workers’ mean of 12 (25) and an oncology nurses’ mean of 10 (9) on avoidance were far lower than all subgroups in Figure 2 except married or cohabiting men not using dolls in treatment and not using therapy dogs [these oncology nurses were more likely to meet the criteria for intrusion or arousal subscale stress than avoidance stress (9)]. In sharp contrast, the other groups in this study had very elevated average avoidance sub-scores of at least 15. However, emergency nurses in Ireland scored even higher on avoidance with averages exceeding 18 (31). High stress also appears in studies of caregivers of family members with Alzheimer’s disease. Association with pets at home tempered the stress of the caregivers that were men or young women, but not for the middle-aged women who had greater family responsibilities (33). Caregivers of family members with dementia in Colombia also experienced high stress; empathy was uniquely associated with this stress (34).

Empathy may have played a role in the elevated avoidance sub-scores of the therapy dog interviewers, with empathy toward the children elevating their stress. Avoidant coping in a study of emergency room nurses was related to somatic complaints (35), where the 248 emergency nurses reported that the most distressing event was dealing with sudden death of young persons. From another study, young adults who had pets (particularly dogs) as children later were more apt to choose work in the helping professions, such as social work, than those who did not have pets during childhood (36). It would take a larger study than this to clarify whether childhood experience with pets was related to electing to work with a dog when interviewing.

The stressed interviewers choosing to use therapy dogs might have been looking to address some of their own stress and discomfort. These dogs are not required to have specific training, only certification as to their temperament and basic obedience skills; arrangements to work with them are relatively simple and expeditious. No responsibility in looking after the dog is involved for the interviewer, since the volunteer owner assumes responsibility for the dogs’ care. This also means the interviewer lacks the special bond with the dog of someone who works and lives with a dog full-time. Regarding the forensic interviewer’s job stress, 75% of all dog handlers felt that the use of a dog in their practice helped to lessen their stress at least somewhat and at most extremely. Almost all facility dog handlers reported they had a lessening of work stress, since incorporating a dog into their practice. Of those using therapy dogs, 57% reported dogs helped with their job stress.

Whether with a facility or therapy dog, during an interview, the child victim’s perceptions and experience would be similar: the child would have a dog to hug, if desired, and offer help through the ordeal. However, the interviewer’s experience differs with a therapy dog that is accompanied by a volunteer and requires special scheduling, than with a facility dog that is a close partner to the interviewer each day, so the effect on the interviewer’s stress may differ in the two situations. Further research would be required to clarify the motivations for choosing each type of dog and the ultimate effects on work stress due to these choices. Having the dog at work all day would have general effects, as well as modifying the interview. More generally, the benefits of dog-friendly work places may manifest as lower rates of absenteeism and higher worker morale and productivity (37).

### Dog Handlers Only

As a whole, most forensic interviewers who incorporated dogs into their practice felt that the dog, whether therapy or facility, was a valuable tool in helping a child cope with the nature of a legal interview, and extremely useful compared to other tools. None of the respondents viewed using a dog as a hindrance. Yet, 28% overall felt that using a dog as a tool was equivalent to using anatomical dolls or drawings.

Facility dog handlers performed almost twice as many interviews per week as therapy handlers; thus, one would expect them to be at heightened risk due to their frequency of interviews (4). The facility dog handlers used the dogs in their interviews more often than those using therapy dogs; since most facility dog handlers felt the dog helped both as a tool to relieve the stress of the victim as well as helping themselves, they may have elected to use a dog more often. Therapy dog users all reported using a dog at most 50% or less of the time. This could be due to feeling that the dog is not always a helpful tool, or simply because a therapy dog is less available, since access needs to be coordinated with the volunteer handler of the dog.

## Conclusion

The standardized testing for secondary stress with the STSS did not support our hypothesis that the use of dogs in forensic interviews would relieve the stress of the interviewers, yet a majority of the interviewers using dogs felt that a dog was an asset to their own mental health in their professional activities. The widespread elevated scores among all interviewer groups on the STSS for avoidance subscores, and the significant differences showing greater stress for the interviewers who had chosen to use a therapy dog as compared with other interviewers, were unexpected. Heightened avoidance sub-scores on the STSS among forensic interviewers have not been reported previously; this suggests a somewhat unique pattern of stress associated with forensic interviewing.

When beginning practice and prior to incorporating a dog into the practice, the facility dog group reported little job stress. The therapy dog group began at a high level of stress that lessened, but continued to be elevated in initial years of interviewing; these interviewers currently still have elevated stress in their professional interviews, as indicated by their elevated STSS avoidance subscale scores, even when adjusting for self-reported stress in the initial interviewing years. This study did not address whether there is a difference between the use of therapy dogs and facility dogs in the degree to which the testimonies from children were made less stressful to the children and more accurate. While lacking such information, this study does suggest that courtroom testimony supervisors may wish to consider expanding facility dog use rather than therapy dog use, on behalf of the interviewers, if dogs are to be used in child testimony proceedings.

## Ethics Statement

The survey was conducted with anonymous and voluntary participation, and the study was approved by the University of California, Davis, Institutional Review Board Protocol #601883-1.

## Author Contributions

DW, MY, and LH conceived and designed the study and collected data. DW and MY prepared the data for analysis. NW, MY, and LH analyzed the data. DW and LH wrote the paper with contributions from MY and NW.

## Conflict of Interest Statement

Pfizer/Zoetis provided a non-designated financial gift for our research in the general area of animal-assisted therapy. The funders had no role in study design, data collection and analysis, decision to publish, or preparation of the manuscript. All authors declare no conflict of interest.
